# Randomised controlled trial of a secondary prevention program for myocardial infarction patients ('ProActive Heart'): study protocol. Secondary prevention program for myocardial infarction patients

**DOI:** 10.1186/1471-2261-9-16

**Published:** 2009-05-09

**Authors:** Anna L Hawkes, John Atherton, C Barr Taylor, Paul Scuffham, Kathy Eadie, Nancy Houston Miller, Brian Oldenburg

**Affiliations:** 1 Viertel Centre for Research in Cancer Control, Cancer Council Queensland, Brisbane, QLD, Australia; 2 Griffith Psychological Health Research Centre, Griffith University, Brisbane, QLD, Australia; 3 School of Public Health and Rehabilitation Sciences, James Cook University, Douglas, QLD, Australia; 4 Royal Brisbane and Women's Hospital, Queensland Health, Brisbane, QLD, Australia; 5 Department of Medicine, University of Queensland, Brisbane, QLD, Australia; 6 Department of Psychology, Stanford University, Palo Alto, California, USA; 7 School of Medicine, Griffith University, Meadowbrook, QLD, Australia; 8 School of Public Health and Preventive Medicine, Monash University, Clayton, Victoria, Australia; 9 Stanford Cardiac Rehabilitation Program, Stanford University, Palo Alto, California, USA

## Abstract

**Background:**

Coronary heart disease (CHD) is a significant cause of health and economic burden. Secondary prevention programs play a pivotal role in the treatment and management of those affected by CHD although participation rates are poor due to patient, provider, health system and societal-level barriers. As such, there is a need to develop innovative secondary prevention programs to address the treatment gap. Telephone-delivered care is convenient, flexible and has been shown to improve behavioural and clinical outcomes following myocardial infarction (MI). This paper presents the design of a randomised controlled trial to evaluate the efficacy of a six-month telephone-delivered secondary prevention program for MI patients (ProActive Heart).

**Methods:**

550 adult MI patients have been recruited over a 14 month period (December 2007 to January 2009) through two Brisbane metropolitan hospitals, and randomised to an intervention or control group (n = 225 per group). The intervention commences within two weeks of hospital discharge delivered by study-trained health professionals ('health coaches') during up to 10 × 30 minute scripted telephone health coaching sessions. Participants also receive a ProActive Heart handbook and an educational resource to use during the health coaching sessions. The intervention focuses on appropriate modification of CHD risk factors, compliance with pharmacological management, and management of psychosocial issues. Data collection occurs at baseline or prior to commencement of the intervention (Time 1), six months follow-up or the completion of the intervention (Time 2), and at 12 months follow-up for longer term outcomes (Time 3). Primary outcome measures include quality of life (Short Form-36) and physical activity (Active Australia Survey). A cost-effective analysis of the costs and outcomes for patients in the intervention and control groups is being conducted from the perspective of health care costs to the government.

**Discussion:**

The results of this study will provide valuable new information about an innovative telephone-delivered cost-effective secondary prevention program for MI patients.

**Trial Registration Number:**

ACTRN12607000595415

## Background

Coronary heart disease (CHD) is a major cause of morbidity, mortality and economic burden in Australia and the rest of the developed world [1]. Secondary prevention programs, with a focus on risk factor management, have been shown to play a pivotal role in the treatment and management of those affected by CHD. The clinical benefits of secondary prevention, or cardiac rehabilitation programs, include decreased total cardiac mortality (26%), improved quality of life (QOL), and lower rates of rehospitalisation [2,3]. As such, guidelines recommend that all persons with CHD participate in secondary prevention programs [4,5].

The traditional and most researched model of secondary prevention consists of participants attending a group outpatient cardiac rehabilitation program for several weeks, including supervised physical activity and CHD risk factor education [6]. However, participation rates in traditional cardiac rehabilitation programs are less than optimal [7]. Witt et al. (2005) reported that in Australia 29% of eligible acute myocardial infarction (MI) patients were referred to cardiac rehabilitation, and only 30% of those referred actually attended, in the United States 29% of MI patients participated, and in Japan only 21% of MI patients participated [7].

Reported barriers to participation in traditional cardiac rehabilitation programs include patient, provider, health system and societal-level barriers such as: older age, female gender, lower education level, a lack of perceived benefit, work or time constraints, transport difficulties, limited availability of programs, lack of reimbursement, as well as limited social or family support [7,8]. Therefore, there is an opportunity and challenge in Australia and internationally to provide innovative CHD services to overcome these barriers to participation, and address the significant treatment gap.

Telephone-delivered interventions are convenient and flexible; they can be delivered at a suitable time for the participant and in their own home; and importantly they improve behavioural outcomes following MI [8,9]. Whilst telephone interventions cannot reach those without access to a telephone, in Australia, approximately 96% of the population live in a household with at least one telephone connection [10]. There are a number of well researched telephone-delivered interventions for CHD patients [11-13] and a large body of literature on home-based and telehealth programs for patients with heart failure [14] or diabetes [15]. Overall, these programs have been shown to be clinically effective, and in a number of cases, cost-effective as well; they also demonstrate high acceptability to participants [16]. We have developed a novel telephone-delivered secondary prevention program that builds on this earlier work to overcome some of the reported barriers to participation in, and adherence to, currently available cardiac rehabilitation programs (ProActive Heart).

We are trialling a state-of-the-art approach to the delivery of secondary prevention for MI patients through: (i) the recruitment and delivery approach used; (ii) the inclusion of a theoretical framework; and (iii) the content of the ProActive Heart program. First, participants are recruited daily by hospital-based staff, to ensure the research team capture all eligible program participants. ProActive Heart is delivered by project-trained and highly skilled health professionals, or 'Health Coaches', over the telephone. The Health Coaches are based off-site which provides flexibility around the translation of ProActive Heart into clinical practice either utilising telehealth lines or helplines available to CHD patients (such as the Heart Foundation's 'Heartline' in Australia) [17] or through acute clinical settings.

Second, theory-based health behaviour interventions are known to be more effective than those that are not theoretically-based [18]. ProActive Heart is grounded in Social Cognitive Theory which has been successfully used across a wide range of health behaviour interventions [18]. ProActive Heart has a focus on the core determinants of health behaviour including: knowledge of the risks and benefits of the behaviour; self efficacy or confidence that one can engage in the behaviour under various circumstances; outcome expectations; and specific strategies for achieving positive health behaviour change [19,20]. Participants develop a personalized action plan (incorporating goal setting) and identification of support networks to enhance behaviour change maintenance. Health Coaches emphasise the benefits of practicing the recommended behaviour, support participants in setting incremental goals to reach the recommended behaviour, with a focus on overcoming self-reported barriers; and provide encouragement to achieve the goal. The intervention targets for addressing individual CHD risk factors are derived from existing national guidelines for the secondary prevention of CHD [5].

Third, in addition to the lifestyle and medical education and support provided by traditional cardiac rehabilitation programs, we have included psychosocial support (with a focus on depression and social isolation) based on the strong and consistent evidence for an independent causal association between depression, social isolation and lack of quality social support and the causes and prognosis of CHD [21].

This paper presents the design of a randomised controlled trial (RCT) to evaluate the efficacy of ProActive Heart to improve the coronary risk factor profile and quality of life of MI patients, as well as cost-effectiveness of the program. We hypothesize that participants in the intervention group will have greater improvements in CHD risk factor behaviours and quality of life, than those in the control group. Additionally, we hypothesise that the ProActive Heart program will be cost-effective compared to the control condition. The results of this study will provide valuable new information about an innovative cost-effective secondary prevention program for CHD patients.

## Methods/Design

### Study Design

The study is a two group prospective RCT in which 550 MI patients are randomised to the intervention (ProActive Heart) or control group. Participants in both groups complete assessments at baseline (Time 1), post-intervention or 6 months follow-up (Time 2), and at 12 months follow-up for longer term effects (Time 3).

### Study Aims

i. To investigate the effects of ProActive Heart on health outcomes [primary outcome variables include quality of life (QOL) and physical activity] post-intervention (Time 2), and at 12 months follow-up (Time 3) for longer term effects.

ii. To examine the cost-effectiveness of ProActive Heart.

### Sample Recruitment Procedures

Ethics approval was received from Human Research Ethics Committees of The Prince Charles Hospital (EC2738), the Royal Brisbane and Women's Hospital (2007/049), and Monash University (2007/0584MC). We have recruited 550 adult MI patients over a 14 month period (December 2007 to January 2009) from two large metropolitan hospitals in Brisbane, Australia (Royal Brisbane and Women's, and The Prince Charles Hospitals). Recruitment staff approached patients in hospital to provide information about the study requirements and assess them for eligibility. Eligible patients who initially agreed to participate were given a description of the study and, if still interested, provided written informed consent, in the presence of a witness, prior to baseline assessment.

Participants were randomly assigned to the intervention or control groups following enrolment (n = 225 in each group). Sample size analysis indicated that 129 subjects per group (intervention and control) or a total of 258 were required to detect, with 90% power and type I error of 5% (two-tailed), an absolute intervention effect of 20% or greater (based on a primary outcome variable of undertaking the recommended level of physical activity [5], of 44% of control and 69% of the intervention over the study period[13]). Sample size was significantly increased above 258 to allow for participant drop-out and subgroup analyses at follow-up. Eligibility criteria included: a diagnosis of MI [typical rise in serum level of troponin with at least one of the following: ischaemic symptoms; development of pathological Q waves on the ECG; ECG changes indicative of ischaemia (ST-segment elevation or depression); or coronary artery intervention] [22]; adults aged 18–80 years; ability to understand English; availability via telephone during the duration of the trial; and no other medical condition that would interfere with optimal participation or produce a significant risk to the patient as defined by the referring specialist.

The intervention commences within the first two weeks of discharge delivered from the Cancer Council Queensland. A letter is mailed to the patient's primary care provider/s informing them of the aims of the study, the patient's agreement to participate and the information that may be required from the patient and the care provider at follow-up. To facilitate comparison of participants and non-participants, de-identified demographic and simple health status data are collected on a sub-sample of eligible MI patients identified by the hospital during the study period, and reasons for refusal are collected by the recruitment staff.

### Study conditions

#### Control

Control participants receive an existing written educational resource ('My Heart My Life')[23]; containing information about CHD and the associated risk factors. 'My Heart My Life' is produced by the Heart Foundation, and provided to all acute coronary syndrome patients in Queensland. They are also sent a quarterly informative newsletter based on existing written educational materials to enhance participant retention.

#### Intervention

Over a six month period, the intervention participants receive up to 10 × 30 minute scripted telephone health coaching sessions from a qualified health professional or 'health coach'. Health coaches are guided by a web-based computer application and key enter all session information. Prior to the commencement of the intervention, participants are posted a ProActive Heart handbook outlining the program goals for CHD risk factors [5], as well as the benefits of improving CHD risk factors. The handbook assists with personal assessment and goal setting that is relevant to the sessions being delivered by the health coach. Participants are also provided with the Heart Foundation educational resource, 'My Heart My Life' [23] and a tape measure for measurement of waist circumference. The participant is encouraged to contact their usual health care providers immediately if there are any medical concerns during the course of the intervention (e.g. changes in cardiac symptoms). Participants requiring additional psychosocial support are triaged and put through to the Helpline based at the Cancer Council Queensland in order to be supported by a trained counsellor.

The health coaching sessions are based on the current guidelines for CHD [5] with a focus on:

1) Appropriate reduction of clinical risk factors (hypercholesterolaemia, hypertension and diabetes),

2) Appropriate modification of behavioural risk factors (smoking, nutrition, alcohol, physical activity, weight management),

3) Compliance with pharmacological management, and

4) Management of psychosocial issues (depression and social isolation).

The sessions include an introductory session to explain the program and what is expected of the participant, followed by three weekly sessions, three fortnightly sessions and four monthly sessions over six months to assist with CHD risk factor management. The frequency of calls decreases over the intervention period to encourage maintenance of behaviour change and self-management. Following the introductory call, the remaining calls are structured as follows: (i) introduction and identification of any cardiac symptom changes; (ii) assessment and health coaching on relevant CHD risk factors; (iii) follow-up on progress towards previous actions and goals; and (iv) session review, including a summary of actions required and scheduling of the next session. Consistent with the self-management approach underpinning the intervention, participants are encouraged to follow-up relevant issues with their usual health care providers. The health coach encourages and recommends the involvement of significant others (i.e. the activation of social and family support), as well as the activation of community and environmental supports to enhance maintenance of behaviour change (e.g. encouraging participants to utilise local gyms or swimming pools).

### Study Integrity

The study design is guided by the CONSORT statement [24], and randomisation to study condition occurs following the completion of Time 1 assessment. Project staff tracking data collection are blinded to condition. Stratified randomisation occurs using a separate block randomisation list that is generated for each subgroup or strata, and randomisation is undertaken by the study manager and concealed from investigators. The schedule is stratified by gender to allow for the expected gender imbalance (70% male, 30% female based on hospital separation data). The intervention protocol is manualised, and all intervention calls are audio-taped with a proportion reviewed to ensure adherence to the delivery of the intervention protocol. All analyses will be conducted on the basis of intention to treat.

### Measurement

Medical or clinical information is collected at Time 1 from hospital medical records (blood pressure, cholesterol level, HbA1c, family history of heart disease, height, weight, body mass index (BMI), procedures done or due, GP details, cardiologist, hospital transferred from, admission and expected discharge date). Additional data is collected at Time 1, Time 2 and Time 3 by computer assisted telephone interview (CATI) using the web-based computer application (see Table 1). Health care utilisation data is validated for 10% of study participants from GP and hospital medical records.

**Table 1:** Measurement of outcome variables at baseline (Time 1), post-intervention (Time 2), and 12 months follow-up (Time 3)

	**Targets based on Heart Foundation guidelines **[5]	**Instrument**
**Primary Outcome Variables**		

Quality of Life	Improvement in Health-related Quality of Life	Short Form 36 [25]

Physical Activity	At least 30 minutes of moderate intensity physical activity on five or more days per week (150 minutes minimum)	Active Australia Survey [29]

**Secondary Outcome Variables**		

Nutrition	Establish/maintain healthy eating patterns, with saturated and trans fatty acid ≤8% of total energy intake	Cancer Council of Victoria Food Frequency Questionnaire (CCVFFQ)[30]

Body mass index (BMI) and waist circumference	Waist measurement ≤ 94 cm (males) or ≤ 80 cm (females) and BMI <25 kg/m^2^	Self Report

Alcohol	Low risk consumption	CCVFFQ

Smoking	Complete cessation Avoid passive smoking	Self report

Psychosocial management	Improvement in depression and decreased social isolation	Hospital Anxiety and Depression Scale [31] ENRICHD Social Support Instrument [32]

Blood Lipids	Total blood cholesterol<4.0 mmol/l; LDL-cholesterol <2.5; HDL-cholesterol > 1.0 mmol/l; Triglycerides < 2.0 mmol/l	Medical records

Blood pressure	Adults≥65: <140/90 mmHg), adults<65: <130/85 mmHg (dependent on age, presence of diabetes, proteinuria and renal insufficiency)	Medical Records

Glycaemic Control	Identify undiagnosed Type 2 diabetes; maintain optimal blood glucose level in those with diabetes (HbA1c^1 ^≤ 7%)	Medical Records

Pharmacological Management	Anti-platelet agents, ACE^2 ^Inhibitors, Beta-blockers, Statins, Anticoagulants Medication Compliance	Medical Records Morisky Medication Compliance Survey [33]

Angina	Controlled Angina	Seattle Angina Questionnaire [34]

1. Glycosylated haemoglobin.

2. Angiotensin converting enzyme inhibitor.

#### Program Implementation

To assess program implementation, we measure satisfaction by self-administered questionnaire at Time 2, and participant adherence to the intervention by data collected from the web-based computer application. Self-administered satisfaction questions include: 'how would you rate the ProActive Heart Handbook' (*excellent *to *poor*); 'how would you rate your sessions with the Health Coach' (*very useful *to *not useful at all*); 'how would you rate the Heart Foundation educational resource My Heart My Life' (*excellent *to *poor*); 'did you get what you expected from the program' (*yes definitely *to *no definitely not*); 'to what extent has ProActive Heart met your needs' (*almost all of my needs have been met *to *none of my needs have been met); *'has ProActive Heart helped you to deal more effectively with your health issues' (*yes it helped a great deal *to *no it seemed to make things worse*); 'in general how satisfied are you with ProActive Heart (*very satisfied *to *quite dissatisfied*); 'were you satisfied with the length of the health coaching sessions' (*yes, no*); 'were you satisfied with the number of health coaching sessions' (*yes, no*); and 'were you satisfied with the length of the intervention overall' (*yes, no*). Participants are also asked to highlight the strengths and weaknesses of ProActive Heart in two open-ended questions. Participant adherence to ProActive Heart is assessed by: the proportion of sessions completed during the intervention period; the topics covered in each session; and the total length (minutes) of intervention exposure during the six month period.

#### Sociodemographics

Self-reported socio-demographic variables include: gender, age, ethnicity, income, education, employment status, and private health insurance status. At follow-up, participants are requested to collect medical information (blood pressure, cholesterol level, HbA1c, BMI) from their primary practitioner medical records prior to CATI.

#### Primary and Secondary Outcome Variables

Primary outcome variables include QOL [25] and physical activity [29]. Secondary outcome variables include: nutrition [30], smoking, BMI, waist circumference, alcohol intake [30], psychosocial management (depression [31] and social support [32]), angina [34], lipid profile, blood pressure, diabetes management (HbA1c), and pharmacological management [33]. The measures for each of these outcome variables are summarised in Table 1.

### Cost-Effectiveness

A cost-effectiveness analysis of the costs and outcomes for patients in the intervention and control groups is conducted from the perspective of health care costs to the government. Detailed self-reported data on costs of health care utilisation (HCU) and medication is collected from both intervention and control groups. HCU data is validated from a randomised sub-sample of 10% of patient records and provider surveys. All resources utilised is multiplied by the appropriate cost using nationally applicable cost data (eg. DRG costs for hospital admissions). The primary health outcome for the cost-effectiveness analyses is quality-adjusted life years (QALYs). These are calculated for both groups using QOL scores from the SF-36 [25] and converted to utility scores using the SF-6D [26]. The measure of relative value for money from the intervention is the incremental cost-effectiveness ratio calculated from the additional cost for a gain in QALY's. A responder analysis is undertaken around the secondary endpoints for the additional cost per responder. Probabilistic and one-way analysis is undertaken around the parameters with uncertainty and/or variability [27].

### Data Analysis

Preliminary analyses establishes the degree of success of the randomisation, and if there are any imbalances of characteristics between the two groups, these are adjusted for in the main analytical modelling. The intervention effect is tested with repeated measures regression models (with baseline scores as co-variates) fitted to estimate changes over time and differences by group in changes over time. A generalised estimating equations approach (SUDAAN statistical package) is used to analyse missing data, accommodating the analysis of partially complete data records. Results are expressed as estimated mean changes in QOL and physical activity outcomes by group as an overall mean excess intervention over usual care effect, all with corresponding 95% confidence intervals. Heirarchical models may also be constructed to describe data collected at different scales (measure, subject, hospital, regional, etc) and incorporate important sources of uncertainty about the parameters of interest, and adjustment for recruitment biases [28].

## Discussion

This study provides critical information on a state-of-the-art telephone delivered and cost-effective secondary prevention program for MI patients. To date, this innovative approach has not been tested by RCT with a cost effectiveness analyses. Further, the study is sufficiently powered to allow for subgroup and secondary analyses. The intervention may be utilised by trained health professionals in a range of settings including broad reach tele-health lines or in acute health settings. As such, the outcomes of this project may be immediately translated in to practice to improve the health outcomes for those affected by CHD.

## Competing interests

The authors declare that they have no competing interests.

## Authors' contributions

ALH and BO developed the study concept and aims and initiated the project. JA, BT, PS, KE and NHM assisted in further development of the study protocol. ALH was responsible for drafting the manuscript. ALH and KE will implement the study protocol and oversee the collection of data. All authors contributed to the final manuscript.

## Pre-publication history

The pre-publication history for this paper can be accessed here:


